# Recent Increase in the Prevalence of Fluconazole-Non-susceptible *Candida tropicalis* Blood Isolates in Turkey: Clinical Implication of Azole-Non-susceptible and Fluconazole Tolerant Phenotypes and Genotyping

**DOI:** 10.3389/fmicb.2020.587278

**Published:** 2020-10-06

**Authors:** Amir Arastehfar, Süleyha Hilmioğlu-Polat, Farnaz Daneshnia, Ahmed Hafez, Mohammadreza Salehi, Furkan Polat, Melike Yaşar, Nazlı Arslan, Tuğrul Hoşbul, Nevzat Ünal, Dilek Yeşim Metin, Şaban Gürcan, Asuman Birinci, Ayşe Nedret Koç, Weihua Pan, Macit Ilkit, David S. Perlin, Cornelia Lass-Flörl

**Affiliations:** ^1^Center for Discovery and Innovation, Hackensack Meridian Health, Nutley, NJ, United States; ^2^Department of Microbiology, Faculty of Medicine, Ege University, Izmir, Turkey; ^3^Westerdijk Fungal Biodiversity Institute, Utrecht, Netherlands; ^4^Biotechvana SL, Valencia, Spain; ^5^Department of Infectious Diseases and Tropical Medicine, Faculty of Medicine, Tehran University of Medical Sciences, Tehran, Iran; ^6^Department of Microbiology, Faculty of Medicine, Dokuz Eylul University, Izmir, Turkey; ^7^Department of Microbiology, Gulhane Training and Research Hospital, University of Health Sciences, Ankara, Turkey; ^8^Division of Mycology, Faculty of Medicine, Çukurova University, Adana, Turkey; ^9^Department of Microbiology, Adana City Hospital, University of Health Sciences, Adana, Turkey; ^10^Department of Microbiology, Faculty of Medicine, Trakya University, Edirne, Turkey; ^11^Department of Microbiology, Faculty of Medicine, Ondokuz Mayis University, Samsun, Turkey; ^12^Department of Microbiology, Faculty of Medicine, Erciyes University, Kayseri, Turkey; ^13^Shanghai Key Laboratory Molecular Medical Mycology, Shanghai, China; ^14^Division of Hygiene and Medical Microbiology, Medical University of Innsbruck, Innsbruck, Austria

**Keywords:** *Candida tropicalis*, antifungal susceptibility testing, genotyping, ERG11, HS1- and HS2-FKS1, candidemia, fluconazole tolerance

## Abstract

*Candida tropicalis* is the fourth leading cause of candidemia in Turkey. Although *C. tropicalis* isolates from 1997 to 2017 were characterized as fully susceptible to antifungals, the increasing global prevalence of azole-non-susceptible (ANS) *C. tropicalis* and the association between high fluconazole tolerance (HFT) and fluconazole therapeutic failure (FTF) prompted us to re-evaluate azole susceptibility of *C. tropicalis* in Turkey. In this study, 161 *C. tropicalis* blood isolates from seven clinical centers were identified by ITS rDNA sequencing, genotyped by multilocus microsatellite typing, and tested for susceptibility to five azoles, two echinocandins, and amphotericin B (AMB); antifungal resistance mechanisms were assessed by sequencing of *ERG11* and *FKS1* genes. The results indicated that *C. tropicalis* isolates, which belonged to 125 genotypes grouped into 11 clusters, were fully susceptible to echinocandins and AMB; however, 18.6% of them had the ANS phenotype but only two carried the ANS-conferring mutation (Y132F). HFT was recorded in 52 isolates, 10 of which were also ANS. Large proportions of patients infected with ANS and HFT isolates (89 and 40.7%, respectively) showed FTF. Patients infected with azole-susceptible or ANS isolates did not differ in mortality, which, however, was significantly lower for those infected with HFT isolates (*P* = 0.007). There were significant differences in mortality (*P* = 0.02), ANS (*P* = 0.012), and HFT (*P* = 0.007) among genotype clusters. The alarming increase in the prevalence of *C. tropicalis* blood isolates with ANS and HFT in Turkey and the notable FTF rate should be a matter of public health concern.

## Introduction

*Candida tropicalis* is the leading cause of candidemia in India, Algeria, and Tunisia (Sellami et al., 2011; Chakrabarti et al., 2014; Megri et al., 2020) and the second to third leading cause of candidemia in South Asian countries (Xiao et al., 2018; Liu et al., 2019). *Candida tropicalis* is associated with a high mortality rate and the poorest prognosis among non-*albicans Candida* (NAC) species (Ko et al., 2019). *Candida tropicalis* has been considered an azole-susceptible NAC species; however, recent studies performed in several countries and worldwide indicate the emergence of azole-resistant *C. tropicalis* isolates in clinical settings (Arendrup et al., 2011; Chakrabarti et al., 2014; Choi et al., 2016; Fan et al., 2018; Xiao et al., 2018; Chen et al., 2019; Pfaller et al., 2019; Megri et al., 2020). According to a SENTRY study, *C. tropicalis*, similar to *C. glabrata*, also demonstrates an increased tendency to echinocandin resistance compared to other *Candida* species, although its rate is lower than that to azoles (Pfaller et al., 2019). Furthermore, a national candidemia study conducted in India indicates the emergence of multidrug-resistant (MDR) *C. tropicalis* isolates in a similar proportion as what observed for MDR *Candida auris* (Chakrabarti et al., 2014). An additional matter of concern is the development of drug tolerance, which may allow the fungus to acquire stable genetic alterations leading to antifungal resistance and which could be potentially linked to azole therapeutic failure and mortality (Rosenberg et al., 2018; Berman and Krysan, 2020). Indeed, several studies indicate that *C. tropicalis* is among the most azole-tolerant *Candida* species (Arthington-Skaggs et al., 2002; Rueda et al., 2017) and that fluconazole efficacy against *C. tropicalis* blood isolates with high azole tolerance is decreased, which can cause fluconazole therapeutic failure (FTF) (Astvad et al., 2018). Considering the limited number of antifungals available to treat candidemia, development of resistance against a single or multiple antifungals should pose a serious threat to patients with candidemia due to *C. tropicalis*, especially in developing countries where fluconazole is the most widely used antifungals (Singh et al., 2018; Arastehfar et al., 2019a,b).

Although our understanding of azole resistance mechanisms in *C. tropicalis* is limited, molecular studies indicate that mutations in the ergosterol biosynthesis gene *ERG11*, which result in reduced affinity of Erg11 to azoles, and/or overexpression of Erg11 and efflux pumps Cdr1 and Mdr1 could be the major contributors to azole resistance (Arastehfar et al., 2020g). At the same time, azole tolerance may depend on changes in the components of stress-response pathways (Rosenberg et al., 2018; Berman and Krysan, 2020). Resistance to echinocandins is mainly caused by mutations in hotspots (HSs) 1 and 2 of the *FKS1* gene encoding the catalytic subunit of β-(1,3)-glucan synthase (Arastehfar et al., 2020g). Genotyping techniques made it possible to determine the link between the genotype and fluconazole resistance and to reveal a propensity of fluconazole-resistant blood isolates of *C. tropicalis* to expand in clusters with a similar genetic background (Chen et al., 2019). Given the association between the genotype and mortality in other *Candida* species (Byun et al., 2018; Arastehfar et al., 2019a), it can be hypothesized that there are similar correlations between azole tolerance and resistance, patient mortality, and the genotype of *C. tropicalis*.

A recent study explored antifungal susceptibility patterns of *Candida* bloodstream isolates in Turkey (1997–2017) and reported the lack of drug resistance for *C. tropicalis* (Arikan-Akdagli et al., 2019). However, our single- and multi-center investigations conducted later in Turkey have revealed a very recent (from 2017 onward) emergence of azole/echinocandin-resistant and even MDR *Candida* species (Arastehfar et al., 2020b,c, e), suggesting the existence of the same trend for *C. tropicalis*. Therefore, we conducted a comprehensive multi-center study to update the rate of antifungal resistance among *C. tropicalis* blood isolates recovered in 2017–2019 in Turkey; some isolates recovered before 2017 but not analyzed in the previous study (Arikan-Akdagli et al., 2019) were also included. The drug resistance mechanisms were explored by sequencing *ERG11* and HS1/2 of *FKS1*. We also examined the level of fluconazole tolerance and assessed its correlation with azole exposure, FTF, and patient mortality. Multilocus microsatellite typing (MMT) was performed to determine the genetic relatedness among *C. tropicalis* bloodstream isolates and associations between genotype clusters and fluconazole minimum inhibitory concentrations (MICs), fluconazole tolerance, and patient outcome.

## Materials and Methods

### Isolation and Identification of *C. tropicalis* and Definition of Clinical Parameters

*Candida tropicalis* bloodstream isolates (*n* = 161) were recovered from 127 patients hospitalized in seven centers: Dokuz Eylül University Hospital (DEUH; *n* = 61), Ege University Hospital (EUH; *n* = 32), Ondokuz Mayıs University Hospital (OMUH; *n* = 22), Trakya University Hospital (TUH; *n* = 17), Erciyes University Hospital (ERUH; *n* = 12), Gülhane Training and Research Hospital (GTRH; *n* = 11), and University of Health Sciences, Adana City Hospital (UHSACH; *n* = 6). Isolates from EUH were collected between 2010 and 2019 but almost 90% of them (29/32) were obtained in 2016–2019. Isolates from DEUH, OMUH, and GTRH were obtained in 2017–2019, those from UHSACH – in 2019, and those from GTRH – in 2013–2015 (with the exception of one isolate obtained in 2019). Of note, isolates collected in DEUH, TUH, and GTRH were not included in the previous Turkish multi-center study (Arikan-Akdagli et al., 2019). Isolates were grown on Sabouraud glucose agar (SGA, Merck, Darmstadt, Germany) and chromogenic media (Candiselect, Bio-Rad, Hercules, CA, United States) at 35°C for 24–48 h. DNA was isolated by a CTAB method (Arastehfar et al., 2018) and species identification was performed by sequencing internal transcribed spacers ITS1 and ITS4 (Stielow et al., 2015). Therapeutic failure was considered if fever persisted and blood culture remained positive (Arastehfar et al., 2020e) despite antifungal treatment and the mortality rate reported herein was all-cause.

### Multilocus Microsatellite Typing

Genotypic diversity of *C. tropicalis* isolates was evaluated according to six markers and 12 loci by using a previously published protocol (Wu et al., 2014). Microsatellite data were organized as categorical values; data analysis was performed by using Bionumerics software v7.6 (Applied Math, Sint-Martens-Latem, Belgium) and dendrograms were constructed according to the unweighted-pair group method using average linkages. Distinct genotypes were considered if two strains differed in more than one locus.

### Antifungal Susceptibility Testing and Tolerance Determination

Antifungal susceptibility testing was performed by using a broth microdilution method recommended by CLSI M27-A3 (Clinical and Laboratory Standards Institute [CLSI], 2008) for the following antifungal drugs: fluconazole, voriconazole, itraconazole, isavuconazole, posaconazole, amphotericin B (AMB) (Sigma-Aldrich, St. Louis, MO, United States), anidulafungin (Pfizer, New York, NY, United States), and micafungin (Astellas, Munich, Germany). MICs were interpreted visually after 24 h incubation at 35°C; *C. parapsilosis* (ATCC 22019) and *C. krusei* (ATCC 6258) strains were used for quality control. Resistance to fluconazole was scored at MICs ≥ 8 μg/ml and that to voriconazole, anidulafungin, and micafungin was considered at MICs ≥ 1 μg/ml (Pfaller and Diekema, 2012). Resistance to posaconazole, itraconazole, and AMB was defined based on epidemiological cut-off values; isolates responding to MICs over 0.12, 0.5, and 2 μg/ml, respectively, were considered non-WT (Pfaller and Diekema, 2012). Since no epidemiological cut-off values and clinical breakpoints were reported for isavuconazole, its MICs were categorized as low (≤0.25 μg/ml) and high (>0.25 μg/ml).

Among the azole antifungals used to treat patients, fluconazole was the main drug applied for both prophylaxis and targeted therapy; therefore, tolerance assessment was performed only for fluconazole. To explore the level of fluconazole tolerance, the mean growth rate above the MIC (4–64 μg/ml) was recorded after 48-h incubation as described previously (Rosenberg et al., 2018; Berman and Krysan, 2020). The level of tolerance was categorized as low-moderate or high when the growth at concentrations above the MIC was <50 and ≥50% compared to the drug-free control, respectively.

### ERG11 and HS1- and HS2-FKS1 Sequencing

*ERG11* and HS1 and HS2 sequencing was performed for all isolates using primers and conditions described previously (Arastehfar et al., 2020a). Contigs were assembled by SeqMan Pro (DNASTAR, Madison, WI, United States) and the resulting sequences were aligned by using MEGA software v7.0 (Kumar et al., 2016) with *C. tropicalis* MYA-3404 genome (AAFN00000000.2) as a WT reference (Butler et al., 2009).

### Data Availability

*ERG11* and HS1- and HS2-*FKS1* sequencing data were deposited in GenBank^1^ under accession numbers MT650724–MT650884, MT650885–MT651045, and MT651046–MT651206, respectively.

### Statistical Analysis

Statistical analysis was performed with SPSS v24 (SPSS Inc., Chicago, IL, United States). Given the high number of genotypes (*n* = 125), we increased the statistical power by grouping isolates into 11 clusters according to genotype similarity. The association between clusters and fluconazole MICs was determined by using chi-square and Kruskal Wallis tests (since the fluconazole MIC data were non-parametric) and the magnitude of association was calculated according to the Eta correlation coefficient. Chi-square test was also used to assess statistical association between the outcome (mortality/survival) and the genotype cluster, and logistic regression analysis was further used at *R* square values over 0.07. Patients infected with multiple isolates of various genotypes were not considered for the association between the genotype cluster and mortality. Correlation between azole tolerance and outcome was assessed by chi-square test. *P* values ≤ 0.05 were considered statistically significant.

## Results

### Clinical Characteristics

A total of 127 patients with the median age of 54 years (range, 2–93 years) were included in the current study; among them, 63% (80/127) were men and 37% (47/127) – women. Most patients were hospitalized in intensive care units (40/127; 31.5%), followed by medical (38/127; 30%), oncology (28/127; 22%), and surgical (21/127; 16.5%) wards. The underlying conditions (some patients had more than one) included solid tumors (36/127; 28.3%), lung diseases (26/127; 20.5%), diabetes mellitus (22/127; 17.3%), hypertension (19/127; 15%), chronic kidney diseases and neurological disorders (18/127; 14.2% each), hematological malignancies and hematopoietic stem cell transplantation (9/127; 7%), and bacterial co-infections (6/127; 4.7%). Given that the final outcome was not available for three patients, the mortality and survival rates were calculated as 54.8% (68/124) and 45.2% (56/124), respectively.

### Multilocus Microsatellite Typing

**FIGURE 1:**
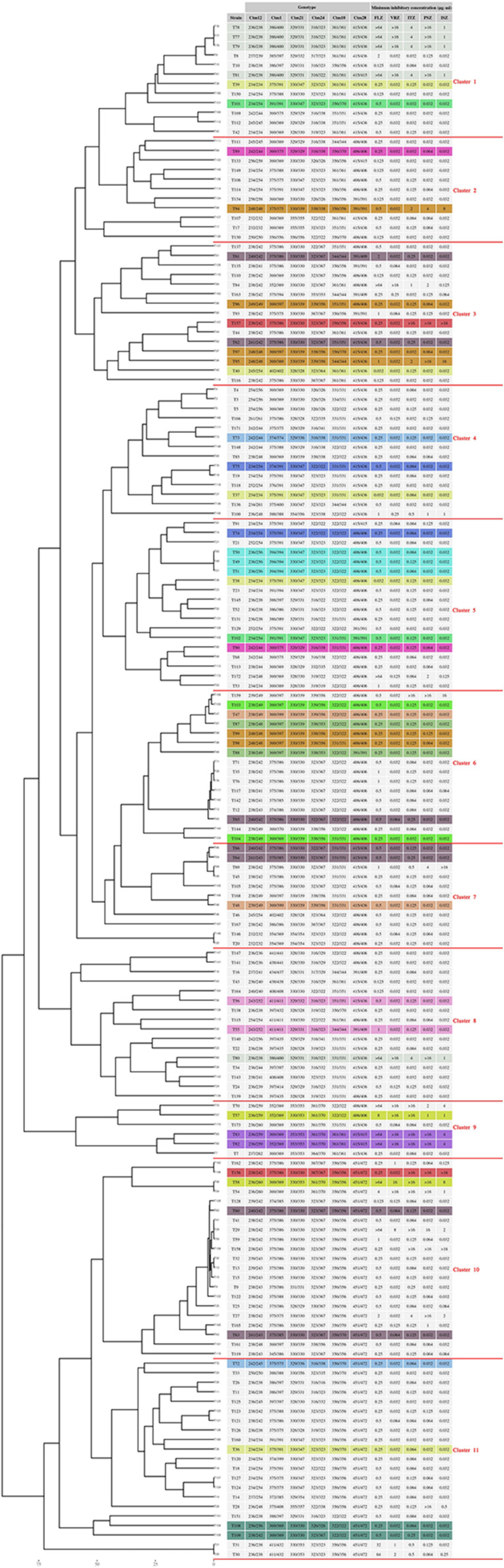
Microsatellite typing of *C. tropicalis* blood isolates collected from seven clinical centers in Turkey. Multiple isolates obtained from the same patient have the same color.

The 161 isolates recovered from the 127 patients belonged to 125 genotypes, which were grouped into 11 clusters (Cs); among them, C5 (18/161; 11.2%), C6 (16/121; 10%), C10 (21/161; 13%), and C11 (21/161; 13%) contained the greatest number of isolates (Figure 1, Supplementary Table S1). Analysis of genotype distribution among hospitals revealed that C10 and C11 isolates were the most prevalent in EUH (46.8%; 15/32), C5–7 and C10 isolates – in DEUH (48.3%; 30/62), and C8, C10, and C11 – in OMUH (7/22; 31.8%), TUH (5/17; 29.4%), and ERUH (4/12; 33.3%) (Supplementary Tables S1, S2). Among repetitive isolates obtained from the same patient, most had the same genotype, with few exceptions (Figure 1, Supplementary Table S1). Logistic regression analysis of correlation between the cluster and patient outcome revealed significant association of C7 with mortality (*P* = 0.02) (Supplementary Material, statistical analysis section, tolerance, and outcome).

### Antifungal Susceptibility Testing

All isolates were susceptible to anidulafungin (≤0.25 μg/ml) and micafungin (≤0.25 μg/ml), and had wild-type (WT) susceptibility to AMB (<2 μg/ml); the azole-non-susceptible (ANS) phenotype was noted for 30 isolates (30/161; 18.6%) obtained from 20 patients (20/127; 15.7%) (Tables 1, 2, Supplementary Table S1). Fluconazole resistance (≥8 μg/ml) and susceptible dose-dependent (4 μg/ml) phenotypes were noted for 15 and 1 isolates (16/161; 10%), respectively, and voriconazole resistance (≥1 μg/ml) and intermediate (0.25–0.5 μg/ml) phenotypes were observed for 16 and 2 isolates (18/161; 11.2%), respectively (Tables 1, 2, Supplementary Table S1). Non-WT susceptibility for itraconazole (>0.5 μg/ml) and posaconazole (>0.12 μg/ml) was revealed for 12.4% (20/161) and 15.5% (25/161) of the isolates, respectively, and elevated MIC of isavuconazole (>0.25 μg/ml) was observed for 13.6% isolates (22/161). The ANS rate varied according to the hospitals and year of isolation. Thus, the ANS phenotype was most frequently observed in TUH (8/17; 47%), followed by DUH (15/61; 24.5%), EUH (5/32; 15.6%), and GTRH (1/11; 9.1%), but was not detected in ERUH, OMUH, and UHSACH. Moreover, 50 and 40% isolates from 2019 recovered in EUH (5/10) and DEUH (12/30), respectively, were ANS. Among the 20 patients infected with ANS isolates, 80% (16/20) were diagnosed in 2018 or later (two-tailed chi-square test, *P* = 0.23). Correlation analysis revealed significant association between genotype clusters and fluconazole MICs (two-tailed chi-square test, *P* = 0.007; Kruskal Wallis test, *P* = 0.012); among the clusters, C1 and C9 were associated with the highest fluconazole MIC (two-tailed chi-square test, *P* = 0.046 and *P* = 0.000, respectively) (Supplementary Material, statistical analysis section, tolerance and outcome). There was no significant difference in mortality between patients infected with ANS and azole-susceptible isolates (two-tailed chi-square test, *P* = 0.987) (Supplementary Material, statistical analysis section, azole resistance and outcome).

**TABLE 1:** Antifungal susceptibility data for *C. tropicalis* blood isolates collected from Turkish hospitals.

AFD	Phenotype	*n*	Minimum inhibitory concentration values (μg/ml)													Range	GM	MIC 50	MIC 90
			≤0.016	0.03	0.06	0.125	0.25	0.5	1	2	4	8	16	32	≥64				
FLC	≤ECV (≤2 μg/ml)	145		3		11	69	50	9	3	1	1		1	13	0.03–≥64	0.54	0.25	2
	>ECV (>2 μg/ml)	16																	
	S (≤2 μg/ml)	145																	
	SDD (4 μg/ml)	1																	
	R (≥8 μg/ml)	15																	
VORI	≤ECV (≤0.06 μg/ml)	138		128	10	5	2		2	1		1	12			0.03–>16	0.06	0.03	0.25
	>ECV (>0.06 μg/ml)	23																	
	S (≤0.12 μg/ml)	143																	
	I (0.25–0.5 μg/ml)	2																	
	R (≥1 μg/ml)	16																	
ITRA	≤ECV (≤0.5 μg/ml)	141		38	40	53	6	4	1	2	6		11			0.03–>16	0.14	0.125	1
	>ECV (>0.5 μg/ml)	20																	
POSA	≤ECV (≤0.12 μg/ml)	136		103	25	8			3	3	2		17			0.03–>16	0.09	0.03	1
	>ECV (>0.12 μg/ml)	25																	
ISA	Low (≤0.25 μg/ml)	139		131	4	3	1	1	8	2	3	2	6			0.03–>16	0.06	0.03	0.5
	High (>0.25 μg/ml)	22																	
ANI	≤ECV (≤0.12 μg/ml)	161	60	48	44	9										0.016–0.125	0.03	0.03	0.06
	>ECV (>0.12 μg/ml)	0																	
	S (≤0.25 μg/ml)	161																	
	I (0.5 μg/ml)	0																	
	R (≥1 μg/ml)	0																	
MICA	≤ECV (≤0.12 μg/ml)	161	152	9												0.016–0.03	0.02	0.016	0.016
	>ECV (>0.12 μg/ml)	0																	
	S (≤0.25 μg/ml)	161																	
	I (0.5 μg/ml)	0																	
	R (≥1 μg/ml)	0																	
AMB	≤ECV (≤2 μg/ml)	161	7			5	22	85	42							0.016–1	0.45	0.5	1
	>ECV (>2 μg/ml)	0																	

*AFD, antifungal drug; FLC, fluconazole; VORI, voriconazole; ITRA, itraconazole; POSA, posaconazole; ISA, isavuconazole; ANI, anidulafungin; MICA, micafungin; AMB, amphotericin B; GM, geometric mean; MIC, minimum inhibitory concentration; ECV, epidemiological cut-off value; R, resistant; S, susceptible; NA, not applicable; MIC ≤ ECV, wild-type; MIC > ECV, non-wild-type.*

**TABLE 2:** Association of azole/fluconazole exposure with the emergence of fluconazole-tolerant and/or azole non-susceptible *C. tropicalis* blood isolates. Patients infected with isolates showing fluconazole tolerance <50% were not included as their clinical data were not available.

	Minimum inhibitory concentration (μg/ml)									
Patient #	Isolate #	FLC	VORI	ITRA	POSA	ISA	Azole prophylaxis	Azole as main treatment	Azole therapeutic failure	Outcome
**Azole non-susceptible isolates (*n* = 20)**										
P1	28	0.25	0.032	0.125	≥16	0.5	None	Yes	FLC failure*	Died
P2	29	≥ 64	8	≥16	16	2	FLC	No (changed to AMB and CASP)	FLC failure	Died
P3	30	64	2	0.5	0.064	0.25	FLC	Yes (VORI changed to CASP)	FLC + VORI failure	Died
P4	82	≥64	≥16	≥16	≥16	4	None	Yes (FLC changed to CASP)	FLC failure	Alive
	83	≥64	≥16	≥16	≥16	4				
P5	172	≥64	0.125	0.064	2	0.125	None	Yes	No	Alive
P6	27	2	0.032	4	≥16	2	None	NO (ANI)	Azole-naïve	Alive
P7	31	32	1	0.5	0.125	0.032	None	NO (MICA)	Azole-naïve	Alive
P8	57	8	≥16	≥16	1	1	None	No (ANI)	Azole-naïve	Alive
	58	≥64	16	≥16	≥16	8				
P9	69	1	0.032	0.5	4	≥16	None	No (MICA)	Azole-naïve	Died
P10	70	≥64	≥16	≥16	2	4	None	No (CASP, MICA, ANI, AMB)	Azole-naïve	Died
P11	77	≥64	≥16	4	≥16	1	None	No (CASP)	Azole-naïve	Died
	78	≥64	≥16	4	≥16	1				
	79	≥64	≥16	4	≥16	1				
	80	≥64	≥16	4	≥16	1				
	81	≥64	≥16	4	≥16	1				
P12	84	≥64	≥16	1	2	0.125	None	No (ANI, AMB)	Azole-naïve	Died
P13	100	1	0.25	0.5	1	1	None	No (CASP)	Azole-naïve	Alive
P14	54	4	≥ 16	≥16	≥16	1		No data	No data	Died
**Azole non-susceptible isolates + fluconazole tolerant isolates (*n* = 10)**										
P15	94	0.5	0.032	2	4	8	None	Yes (FLC changed to MICA)	FLC failure	Died
	95	1	0.032	2	≥ 16	16				
	96	0.25	0.032	0.125	0.064	0.032				
	97	0.25	0.032	0.032	0.064	0.032				
P16	156	0.25	0.032	≥ 16	≥16	≥16	None	Yes (FLC changed to ANI)	FLC failure	Alive
	157	0.25	0.032	≥ 16	≥ 16	≥16				
P17	158	0.25	0.032	≥ 16	≥16	≥16	None	Yes (FLC changed to ANI)	FLC failure	Died
P18	165	0.25	0.125	0.125	1	0.032	None	Yes (FLC changed to ANI)	FLC failure	Alive
P19	159	0.5	0.032	≥ 16	≥16	16	None	None	Azole-naïve	Died
P20	162	0.25	1	0.125	0.064	0.125	None	No (ANI)	Azole-naïve	Alive
**Fluconazole-tolerant isolates exposed to azoles (*n* = 23)**										
23 patients	23 isolates	0.25–1	0.032	0.032–125	0.032–0.125	0.32	Yes (*n* = 2)	Yes (*n* = 23)	Yes (*n* = 7)**	Died (*n* = 7)
							No (*n* = 21)		No (*n* = 16)	Alive (*n* = 16)
**Fluconazole-tolerant isolates not exposed to azoles (*n* = 18)**										
18 patients	19 isolates	0.25–0.5	0.32–0.064	0.032–0.125	0.032–0.064		None	No (*n* = 14)	NA	Died (*n* = 9)
								No treatment (*n* = 4)		Alive (*n* = 9)

* This patient had persisting fever and died while being treated with fluconazole. ** Treatment changed to CASP (*n* = 1), AMB + ANI (*n* = 1), ANI (*n* = 1), VORI (*n* = 1), AMB (*n* = 1), or MICA (*n* = 1); among whom only three died. FLC, fluconazole; VORI, voriconazole; ITRA, itraconazole; POSA, posaconazole; ISA, isavuconazole; AMB, amphotericin B; ANI, anidulafungin; MICA, micafungin; CASP, caspofungin.

### ERG11 and HS1- and HS2-FKS1 Sequencing

Among the *C. tropicalis* isolates, 12% (19/161) harbored non-synonymous mutations in *ERG11*; V362I (*n* = 12), R245K (*n* = 10), and Y221F (*n* = 7) were the most frequently observed amino acid substitutions, followed by K344N (*n* = 6), K344T and V326M (*n* = 4 each), and S154F and Y132F (*n* = 2 each). Among the ANS isolates, only two (# 30 and #31) had *ERG11* mutations (Y132F + S154F) (Supplementary Table S1). None of the isolates had mutations in HS1 and HS2 of *FKS1*, which is consistent with the echinocandin susceptibility results.

### Fluconazole Tolerance

Tolerance to fluconazole was identified in 33.5% isolates (54/161), among which 96.2% (52/54) showed high fluconazole tolerance (HFT) (≥50% growth at concentrations over the MIC) (Table 2, Supplementary Table S1). These 54 isolates were recovered from 49 patients and clinical outcome was not available for patients infected with isolates showing low to moderate fluconazole tolerance (<50% growth at concentrations over the MIC) (Table 2). Among the hospitals, ERUH (12/12; 100%), TUH (15/17; 88.2%), and UHSACH (5/6; 83.3%) had the highest rates of isolates with HFT, followed by OMUH (7/22; 31.8%), EUH (7/32; 21.8%), and DEUH (6/61, 9.8%; 2 isolates had <50% tolerance: 2/61; 3.2%), whereas none of the isolates from GTRH showed fluconazole tolerance. The mortality rate was significantly higher in patients infected with *C. tropicalis* isolates without fluconazole tolerance compared to those infected with HFT isolates [49/77 vs 19/47 (no outcome information for three patients); two-tailed chi-square test, *P* = 0.007] (Supplementary Material, statistical analysis section, tolerance and outcome). We also observed a significant difference in HFT distribution among the clusters (two-tailed chi-square test, *P* = 0.003); thus, C5 and C8 had the greatest numbers of fluconazole-tolerant isolates (two-tailed chi-square test, *P* = 0.045 and *P* = 0.006, respectively), whereas C3 had the smallest number of HFT isolates (two-tailed chi-square test, *P* = 0.014).

### Azole Exposure and FTF Among Patients Infected With Fluconazole-Tolerant and ANS Isolates

Patients were divided into three categories depending on isolate tolerance and azole non-susceptibility profiles: ANS (*n* = 14), ANS-HFT (*n* = 6) (note that these isolates were fluconazole susceptible), and HFT (*n* = 41) (Table 2); two patients with low to moderate fluconazole tolerance were not included because of unavailable clinical data. Among the 14 patients infected with ANS isolates, eight were azole-naïve, one did not have treatment data, and five received fluconazole, of which 80% (4/5) showed FTF (4/5) (Table 2). Among the patients infected with ANS-HFT isolates, four were treated with fluconazole and showed FTF (4/6; 66.6%) and two were azole-naïve (2/6; 33.4%). Among the 41 patients infected with HFT isolates, 18 (44%) were azole-naïve and the rest received azoles (56%), of which 30.4% (7/23; 30.4%) showed FTF (Table 2). Statistical analysis indicated that patients infected with ANS and ANS-HFT isolates had higher FTF (*P* = 0.046 and *P* = 0.023, respectively) than those infected with HFT isolates (*P* = 0.103) and that the ANS and ANS-HFT phenotypes were associated with FTF (logistic regression coefficients 2.213 vs 22.030) (see Supplementary Material, statistical analysis section, therapeutic failure).

## Discussion

In this study, we revealed a considerable rate of infection with ANS *C. tropicalis* in Turkey (15.7%), which is in contrast with a previous multi-center study, which did not detect any such isolates (0%) (Arikan-Akdagli et al., 2019). We also observed that HFT and ANS isolates could be encountered in azole-naïve as well as in fluconazole-treated patients and that ANS and HFT phenotypes, although with varying degrees, were associated with FTF. Analysis of microsatellite cluster distribution revealed its significant association with fluconazole MICs and tolerance, and patient mortality, which highlights the clinical importance of genotyping data.

Consistent with previous study in Turkey (Arikan-Akdagli et al., 2019), the antifungal susceptibility profile of *C. tropicalis* updated in this study indicated the absence of isolates with echinocandin resistance and/or non-WT susceptibility to AMB. In contrast to a previous study reporting the absence of azole-resistant *C. tropicalis* (Arikan-Akdagli et al., 2019), however, we identified 18.6% ANS *C. tropicalis* isolates, among which 93 and 58% were cross-resistant to three azoles or fluconazole + voriconazole, respectively. Importantly, we observed an alarming rate (40–50%) of ANS isolates in some hospitals in 2019, which is also documented in other studies (Arendrup et al., 2011; Chen et al., 2019; Pfaller et al., 2019); these statistics require particular attention, especially in the countries where azoles are the main antifungals used for candidemia therapy (Sellami et al., 2011; Singh et al., 2018; Arastehfar et al., 2019a, 2020e). Notable variations in the rate of azole-resistant isolates (0–100%) observed among the analyzed hospitals could be attributed to differences in intervention strategies, including the use of azoles and infection control practices. To address the resistance mechanism, we sequenced the *ERG11* gene and unexpectedly found a very low rate of the Y132F mutation considered responsible for azole resistance (Fan et al., 2018), which was identified in only two azole-resistant isolates. However, many other factors can contribute to azole resistance (Fan et al., 2018; Arastehfar et al., 2020g); thus, a recent study did not identify accountable *ERG11* mutations in fluconazole-resistant *C. tropicalis* blood isolates but instead found specific polymorphisms in transcription factors regulating the expression of *ERG11* and efflux pump-related genes, i.e., *UPC2*, *MRR1*, and *TAC1* (Arastehfar et al., 2020a). Although we detected other *ERG11* mutations, they were found exclusively in azole-susceptible isolates, which is consistent with a previous report (Oliveira et al., 2013).

To understand the association between azole use and the emergence of ANS *C. tropicalis* isolates, we analyzed the clinical history of respective patients (20/127; 15.7%) and found that 50% of them were azole-naïve, which is similar to the results obtained for patients infected with *Candida parapsilosis* (Tóth et al., 2019) and *C. auris* (Jeffery-Smith et al., 2018). Recent studies from Iran (Arastehfar et al., 2020a), Taiwan (Chen et al., 2019), and Japan (Chong et al., 2012) have reported the same phenomenon, which could be the driving force behind the replacement of *C. albicans* as the first leading cause of candidemia with *C. tropicalis* documented in Taiwan (Tulyaprawat et al., 2020). We also found an association between fluconazole resistance and genotypes, which may account for the dramatic increase in the number and persistence of ANS isolates in clinical settings since the previous candidemia study in Turkey (Arikan-Akdagli et al., 2019), hypothetically because azole-resistant isolates may possess a higher mutagenesis potential allowing them to thrive in conditions when fluconazole is heavily used (Healey et al., 2016). The remaining ANS isolates were recovered from nine patients, almost 89% of them (8/9) showed FTF. Regarding the two patients infected with the *C. tropicalis* isolates harboring Y132F, one was azole-naïve (#30) and the other one in keeping with the other *Candida* species carrying Y132F (Arastehfar et al., 2020g) showed failure to both fluconazole and voriconazole for which the isolate was resistant against both agents (#30).

Although we did not detect echinocandin resistance and all *C. tropicalis* isolates studied had WT for *FKS1*, considering the global increase in echinocandin-resistant *C. tropicalis* (Pfaller et al., 2019), the emergence of such isolates in Turkey could be only a matter of time, especially in view of a significant increase in ANS isolates over the past few years observed in this study.

Analysis of fluconazole tolerance, which is known to be considerable among *C. tropicalis* isolates, revealed a lower tolerance rate (34%) compared to a previous study (73.5%) (Rueda et al., 2017); however, 96.2% of our isolates were HFT. Similar to patients infected with ANS isolates, a notable proportion (44%) of the patients infected with fluconazole-tolerant isolates were azole-naïve. Among the patients infected with fluconazole-tolerant *C. tropicalis* and treated with fluconazole/azoles, approximately 31% showed FTF; furthermore, all patients (100%) infected with HFT-ANS isolates had FTF. Although pharmacodynamics and other host-related factors affecting therapeutic success should be considered, our findings, which are consistent with earlier studies (Astvad et al., 2018; Rosenberg et al., 2018), highlight the importance of fluconazole tolerance assessment in clinical settings. We propose that *C. tropicalis* isolates exhibiting HFT (≥50– >75% growth at concentrations over MIC) may predict azole therapeutic failure. Indeed, the clinical implication of tolerance in the bacteriology field is more appreciated, where it has been associated with prolonged antibiotic treatment, therapeutic failure, and rapid development of resistance (Levin-Reisman et al., 2017; Windels et al., 2019; Berti and Hirsch, 2020; Liu et al., 2020). More importantly, antibiotic tolerance can threaten the clinical efficacy of combination antibiotic therapy used to minimize the emergence of resistant bacteria and to enhance the efficacy of treatment (Liu et al., 2020). Similarly, emerging recent studies in medical mycology documented FTF among isolates showing high level of tolerance to this drug (Astvad et al., 2018; Rosenberg et al., 2018). Altogether, these studies point to the importance of measurement of tolerance among clinically obtained bacterial and fungal isolates to guide clinicians in choosing an appropriate antifungal regimen. Of note, collecting multiple azole susceptible *C. tropicalis* isolates with undetected azole tolerance from the same patients might be attributable to host-related factors rendering them susceptible to acquire persistent candidemia (Arastehfar et al., 2020g,f).

In agreement with a previous study (Rueda et al., 2017), we found that patients carrying HFT isolates had a significantly lower mortality rate than those carrying isolates without detectable tolerance, which could be associated with unknown genomic mutations affecting the fitness cost (Rueda et al., 2017; Rosenberg et al., 2018). In contrast, we did not find significant difference in mortality between patients infected with azole-resistant and azole-susceptible isolates, which could be due to a negligible or no fitness cost for acquiring drug resistance. *In vivo* testing is needed to verify this hypothesis.

Although a previous study has not revealed correlation between the genotype and fluconazole tolerance, it could be attributed to a limited number of genotyped *C. tropicalis* isolates: each of 46 identified genotypes was represented with only a single isolate (Marcos-Zambrano et al., 2016), which could have prevented establishing a statistically significant link. In this study, we genotyped 161 *C. tropicalis* isolates and, to increase the statistical power of correlation analysis, grouped them to clusters containing related genotypes, which resulted in finding an association between genotype clusters and fluconazole tolerance. Interestingly, the clusters significantly differed in the number of HFT isolates, which could be explained by genetic, physiological, and metabolic variations among the strains (Rosenberg et al., 2018; Berman and Krysan, 2020). We also revealed correlation between the genotype cluster and patient mortality, which has been previously shown for *C. glabrata* (Byun et al., 2018; Arastehfar et al., 2019a). The mortality rate observed in this study is similar to those reported for *C. tropicalis* in Iran (Arastehfar et al., 2020a), Italy (Montagna et al., 2013), and the United States (Andes et al., 2016), and to that observed for *C. glabrata* in Turkey (Arastehfar et al., 2020e). Collectively, these findings emphasize the importance of using genotyping techniques in clinical settings, which may provide insightful observations with predictive prognostic values.

This study had some limitations, the main of which was the lack of detailed treatment data (dosage and duration) and clinical information due to the retrospective nature of the study. Also, we did not examine the expression of efflux pumps and *ERG11* or sequence their master regulator genes *MRR1*, *TAC1*, and *UPC2*, which will be the scope of future research. Lastly, the mutation prevention concentration experiments, which quantitatively explores the azole tolerance is lacking in this study.

In summary, the current study revealed notable and rapid emergence of ANS isolates in Turkey, which necessitates continuous monitoring of the antifungal resistance rate on a multi-center scale. Furthermore, our results point on the clinical utility of azole susceptibility/tolerance profiling and genotyping, which can provide clues regarding azole therapeutic failure or success.

## ORCID

Amir Arastehfar

orcid.org/0000-0002-4361-4841

Süleyha Hilmioğlu-Polat

orcid.org/0000-0001-8850-2715

Farnaz Daneshnia

orcid.org/0000-0002-8782-2036

Furkan Polat

orcid.org/0000-0003-4640-3991

Melike Yaşar

orcid.org/0000-0001-8913-2314

Nazlı Arslan

orcid.org/0000-0002-3951-4418

Tuğrul Hoşbul

orcid.org/0000-0002-0150-4417

Nevzat Ünal

orcid.org/0000-0001-5121-3100

Dilek Yeşim Metin

orcid.org/0000-0002-7282-5031

Şaban Gürcan

orcid.org/0000-0002-5052-481X

Asuman Birinci

orcid.org/0000-0002-8653-4710

Nedret Koç

orcid.org/0000-0002-1736-9707

Macit Ilkit

orcid.org/0000-0002-1174-4182

Cornelia Lass-Flörl

orcid.org/0000-0002-2946-7785

David S. Perlin

orcid.org/0000-0002-9503-3184

## Conclusion

*Candida tropicalis* is the fourth leading cause of candidemia in Turkey and the blood isolates recovered during 1997–2017 were entirely susceptible to all antifungals tested. The studies conducted in other countries, however, have shown a dramatic increase in the number of ANS *C. tropicalis* blood isolates. Moreover, *C. tropicalis* proved to be the most tolerant *Candida* species to fluconazole and those with HFT showed FTF when tested *in vivo*. Therefore, the scope of our study was to reevaluate the burden of antifungal resistance of *C. tropicalis* blood isolates on a multicenter scale in Turkey and to evaluate the influence of ANS and HFT on fluconazole exposure, FTF, and mortality. Moreover, we sequenced the HS and HS2 of FKS1 and *ERG11* to evaluate the echinocandins and azole resistance mechanisms. Indeed, as expected we observed a significant increase in the number of ANS isolates from 2017 onward when compared to the previous study conducted in Turkey. Interestingly, patients infected with both ANS and HFT *C. tropicalis* blood isolates showed FTF and some microsatellite clusters significantly had a higher number of ANS and HFT isolates. Similarly, we observed that some clusters were associated with a higher mortality rate. Collectively, our study implicated the growing concern of ANS *C. tropicalis* blood isolates in Turkey and showed that HFT isolates, similar to ANS isolates, can cause FTF. Moreover, we showed that the application of genotyping techniques may have prognostic values with regards to mortality and FTF in candidemia settings.

## Data Availability Statement

The datasets presented in this study can be found in online repositories. The names of the repository/repositories and accession number(s) can be found in the article/ Supplementary Material.

## Ethics Statement

This study was approved by Ege University Hospital Ethical Committee and was granted with the following ethical approval number (20-2T/30). Consent forms were obtained from patients and we used numerical codes for *C. tropicalis* isolates to anonymize the patient’s identity. Written informed consent to participate in this study was provided by the participants’ legal guardian/next of kin.

## Author Contributions

AA, WP, MI, DP, and CL-F: conceptualization and methodology. AA, FD, AH, and MS: software. AA, FD, SH-P, and CL-F: validation. AA, FD, WP, MI, SH-P, and CL-F: formal analysis. AA, SH-P, FD, AH, MS, FP, MY, NA, TH, NÜ, DM, SG, AK, WP, MI, and CL-F: investigation. SH-P, FP, MY, NA, TH, NÜ, DM, SG, AK, MI, WP, and CL-F: resources. AA, SH-P, FD, AH, MS, and CL-F: data curation. AA: writing – original draft preparation. AA, SH-P, FD, AH, MS, FP, MY, NA, TH, NÜ, DM, SG, AK, WP, MI, DP, and CL-F: writing – review and editing. AA, AH, and MS: visualization. AA, SH-P, MI, WP, and CL-F: supervision. AA, SH-P, DP, CL-F: project administration. WP, SH-P, MI, and CL-F: funding acquisition. All authors have read and agreed to the published version of the manuscript.

## Conflict of Interest

The authors declare that the research was conducted in the absence of any commercial or financial relationships that could be construed as a potential conflict of interest.

## Footnotes

1 https://www.ncbi.nlm.nih.gov/genbank/

## Supplementary Material

Supplementary Table S1 Antifungal susceptibility, genotypes, Fks1p/Fks2p and Erg11p mutations, and clinical outcomes of patients infected with *C. tropicalis* isolates.

Supplementary Table S2 Distribution of genotype clusters among seven hospitals participated in this study.
