# Supplementary material for: Recent Increase in the Prevalence of Fluconazole-Non-susceptible Candida tropicalis Blood Isolates in Turkey: Clinical Implication of Azole-Non-susceptible and Fluconazole Tolerant Phenotypes and Genotyping
Source: Front Microbiol. 2020 Oct 6;11:587278. doi: 10.3389/fmicb.2020.587278 (PMC7573116; doi:10.3389/fmicb.2020.587278)
Supplement: Supplementary Table S1 — Antifungal susceptibility, genotypes, Fks1p/Fks2p and Erg11p mutations, and clinical outcomes of patients infected with C. tropicalis isolates. [file Data_Sheet_1.pdf]

TABLE S1 | Antifungal susceptibility, genotypes, Fks1p/Fks2p and Erg11p mutations, and clinical outcomes of patients infected with *C. tropicalis* isolates.

				Minimum inhibitory concentration (µg/ml)									
**Strain #**	**Hospital**	**Cluster/Genotype**	**Tolerance level**	**FLZ**	**VRZ**	**ITZ**	**PSZ**	**ISZ**	**AND**	**MICA**	**AMB**	**Erg11p**	**Outcome**
**3**	Ege University Hospital	C4/ G34	≥50%	0.25	0.032	0.032	0.032	0.032	0.064	0.016	1	K344T+V362I	Survived
**4**	Ege University Hospital	C4/ G34	≥50%	0.25	0.032	0.064	0.032	0.032	0.032	0.016	1	K344N+V362M	Died
**5**	Ege University Hospital	C4/ G34	≥50%	0.25	0.032	0.125	0.032	0.032	0.032	0.016	1	K344T+V362I	Died
**7**	Ege University Hospital	C9/ G94	No	0.25	0.032	0.064	0.032	0.032	0.016	0.016	1	WT	Died
**8**	Ege University Hospital	C1/ G2	No	2	0.032	0.032	0.125	0.032	0.032	0.016	1	WT	Survived
**9**	Ege University Hospital	C10/ G101	No	0.25	0.032	0.25	0.032	0.032	0.064	0.016	1	WT	Died
**10**	Ege University Hospital	C1/ G3	No	0.125	0.032	0.064	0.032	0.032	0.032	0.016	1	WT	Survived
**11**	Ege University Hospital	C11/ G111	No	0.25	0.032	0.125	0.032	0.032	0.016	0.016	1	WT	Survived
**12**	Ege University Hospital	C6/ G63	No	0.5	0.032	0.064	0.032	0.032	0.016	0.016	1	WT	Died
**13**	Ege University Hospital	C10/ G100	≥50%	0.5	0.032	0.064	0.032	0.032	0.064	0.016	1	WT	Survived
**14**	Ege University Hospital	C11/ G120	No	0.25	0.032	0.064	0.032	0.032	0.064	0.016	1	WT	Died
**15**	Ege University Hospital	C10/ G100	No	0.5	0.032	0.125	0.032	0.032	0.064	0.016	1	WT	Died
**16**	Ege University Hospital	C8/ G77	No	0.25	0.032	0.064	0.032	0.032	0.016	0.016	1	WT	Died
**17**	Ege University Hospital	C2/ G18	No	0.5	0.032	0.125	0.064	0.032	0.032	0.016	1	Y221F+R245K+K344T+V362I	Died
**18**	Ege University Hospital	C11/ G118	No	0.5	0.032	0.064	0.032	0.032	0.032	0.016	0.5	WT	Survived
**19**	Ege University Hospital	C4/ G41	No	0.25	0.032	0.064	0.032	0.032	0.064	0.016	0.5	WT	Survived
**20**	Ege University Hospital	C7/ G75	No	0.25	0.032	0.125	0.032	0.032	0.016	0.016	0.5	R245K+K344N+V362I	Survived
**21**	Ege University Hospital	C5/ G47	No	0.5	0.032	0.064	0.032	0.032	0.016	0.016	0.5	WT	Died
**22**	Ege University Hospital	C8/ G85	≥50%	0.25	0.032	0.064	0.032	0.032	0.064	0.016	0.5	WT	Died
**23**	Ege University Hospital	C5/ G49	No	0.5	0.032	0.125	0.032	0.032	0.032	0.016	0.5	WT	Died
**24**	Ege University Hospital	C8/ G89	No	0.5	0.125	0.125	0.032	0.032	0.032	0.016	1	WT	Died
**25**	Ege University Hospital	C10/ G103	No	0.5	0.032	0.064	0.032	0.064	0.032	0.016	1	WT	Survived
**26**	Ege University Hospital	C11/ G111	≥50%	0.25	0.032	0.064	0.032	0.032	0.032	0.016	0.5	WT	Survived
**27**	Ege University Hospital	C10/ G104	No	2	0.032	4	>16	2	0.064	0.016	1	WT	Survived
**28**	Ege University Hospital	C11/ G121	No	0.25	0.032	0.125	>16	0.5	0.016	0.016	1	WT	Died
**29**	Ege University Hospital	C10/ G99	No	>64	8	>16	16	2	0.032	0.032	1	WT	Died
**30**	Ege University Hospital	C11/ G125	No	64	2	0.5	0.064	0.25	0.032	0.016	1	Y132F+S154F	Died
**31**	Ege University Hospital	C11/ G125	No	32	1	0.5	0.125	0.032	0.032	0.016	0.5	Y132F+S154F	Survived

**32**	Ege University Hospital	C10/ G100	No	0.25	0.032	0.125	0.032	0.032	0.032	0.016	0.5	WT	Survived
**33**	Ege University Hospital	C11/ G110	No	0.25	0.032	0.032	0.032	0.032	0.016	0.016	0.5	WT	Died
**34**	Ege University Hospital	C8/ G87	No	0.25	0.032	0.032	0.032	0.032	0.016	0.016	0.5	WT	Survived
**35**	Ege University Hospital	C6/ G61	≥50%	1	0.032	0.125	0.032	0.032	0.016	0.016	0.5	WT	Survived
**36**	Dokuz Eylül University Hospital	C11/ G116	No	0.25	0.032	0.064	0.032	0.032	0.06	0.016	0.5	WT	Survived
**37**	Dokuz Eylül University Hospital	C4/ G43	No	0.032	0.032	0.064	0.032	0.032	0.06	0.016	0.5	WT	
**38**	Dokuz Eylül University Hospital	C5/ G49	No	0.032	0.032	0.125	0.032	0.032	0.06	0.016	0.5	WT	
**39**	Dokuz Eylül University Hospital	C1/ G5	No	0.25	0.032	0.125	0.032	0.032	0.064	0.016	1	WT	
**40**	Dokuz Eylül University Hospital	C3/ G32	≥50%	0.032	0.032	0.125	0.032	0.032	0.032	0.016	0.5	WT	
**41**	Dokuz Eylül University Hospital	C10/ G99	No	0.5	0.032	0.064	0.032	0.032	0.032	0.016	1	WT	Died
**42**	Dokuz Eylül University Hospital	C1/ G9	No	0.5	0.032	0.125	0.032	0.032	0.032	0.016	1	WT	Survived
**43**	Dokuz Eylül University Hospital	C8/ G78	≥50%	0.125	0.032	0.032	0.032	0.032	0.064	0.016	0.5	WT	Survived
**44**	Dokuz Eylül University Hospital	C3/ G28	No	0.25	0.032	0.125	0.032	0.032	0.032	0.016	1	WT	Survived
**45**	Dokuz Eylül University Hospital	C7/ G69	No	0.25	0.032	0.125	0.032	0.032	0.016	0.016	1	WT	Died
**46**	Dokuz Eylül University Hospital	C7/ G73	No	0.5	0.032	0.125	0.032	0.032	0.032	0.016	0.5	WT	Died
**47**	Dokuz Eylül University Hospital	C6/ G56	No	0.25	0.032	0.125	0.032	0.032	0.032	0.016	0.5	WT	Survived
**48**	Dokuz Eylül University Hospital	C7/ G72	No	0.5	0.032	0.125	0.032	0.032	0.032	0.016	0.5	WT	
**49**	Dokuz Eylül University Hospital	C5/ G48	No	0.5	0.032	0.125	0.032	0.032	0.064	0.016	0.5	WT	Died
**50**	Dokuz Eylül University Hospital	C5/ G48	No	0.5	0.032	0.064	0.032	0.032	0.064	0.016	0.5	WT	
**51**	Dokuz Eylül University Hospital	C5/ G48	No	0.5	0.032	0.064	0.032	0.032	0.064	0.016	0.5	WT	
**52**	Dokuz Eylül University Hospital	C5/ G50	No	0.5	0.032	0.125	0.032	0.032	0.064	0.016	0.5	WT	Died
**53**	Dokuz Eylül University Hospital	C5/ G55	<50%	1	0.032	0.125	0.032	0.032	0.064	0.032	0.5	WT	No data
**54**	Dokuz Eylül University Hospital	C10/ G96	No	4	>16	>16	>16	1	0.032	0.016	1	WT	Died
**55**	Dokuz Eylül University Hospital	C8/ G83	No	1	0.032	0.125	0.032	0.032	0.064	0.016	1	WT	Died
**56**	Dokuz Eylül University Hospital	C8/ G80	No	0.5	0.032	0.125	0.032	0.032	0.125	0.016	1	WT	
**57**	Dokuz Eylül University Hospital	C9/ G91	No	8	>16	>16	1	1	0.064	0.016	1	WT	Survived
**58**	Dokuz Eylül University Hospital	C10/ G96	No	>64	16	>16	>16	8	0.016	0.016	1	WT	
**59**	Dokuz Eylül University Hospital	C10/ G99	<50%	1	0.032	0.125	0.064	0.032	0.032	0.016	1	WT	Survived
**60**	Dokuz Eylül University Hospital	C10/ G98	No	0.5	0.064	0.125	0.032	0.032	0.032	0.016	1	WT	Died
**61**	Dokuz Eylül University Hospital	C3/ G21	No	2	0.032	0.25	0.032	0.032	0.032	0.016	0.5	WT	
**62**	Dokuz Eylül University Hospital	C3/ G29	No	0.5	0.032	0.25	0.032	0.032	0.064	0.016	0.5	WT	
**63**	Dokuz Eylül University Hospital	C10/ G106	No	0.5	0.064	0.125	0.032	0.032	0.064	0.016	0.5	WT	

**64**	Dokuz Eylül University Hospital	C7/ G68	No	0.5	0.032	0.25	0.032	0.032	0.064	0.016	0.5	WT	
**65**	Dokuz Eylül University Hospital	C6/ G64	No	0.5	0.064	0.25	0.032	0.032	0.125	0.016	0.5	WT	
**66**	Dokuz Eylül University Hospital	C7/ G67	No	0.5	0.032	0.125	0.032	0.032	0.125	0.016	0.5	WT	
**68**	Dokuz Eylül University Hospital	C5/ G53	No	0.25	0.032	0.064	0.032	0.032	0.064	0.032	0.5	WT	Died
**69**	Dokuz Eylül University Hospital	C7/ G69	No	1	0.032	0.5	4	>16	0.032	0.016	0.5	WT	Died
**70**	Dokuz Eylül University Hospital	C9/ G91	No	>64	>16	>16	2	4	0.032	0.016	0.5	WT	Died
**71**	Dokuz Eylül University Hospital	C6/ G61	No	0.5	0.032	0.064	0.032	0.032	0.032	0.016	0.5	WT	Died
**72**	Dokuz Eylül University Hospital	C11/ G109	No	0.25	0.032	0.064	0.032	0.032	0.064	0.016	0.5	WT	Survived
**73**	Dokuz Eylül University Hospital	C4/ G37	No	0.25	0.032	0.125	0.032	0.032	0.032	0.016	0.5	WT	
**74**	Dokuz Eylül University Hospital	C5/ G46	No	0.25	0.032	0.064	0.032	0.032	0.032	0.016	0.5	WT	Survived
**75**	Dokuz Eylül University Hospital	C4/ G40	No	0.5	0.032	0.064	0.032	0.032	0.064	0.016	0.5	WT	
**76**	Dokuz Eylül University Hospital	C6/ G61	No	1	0.032	0.125	0.032	0.032	0.064	0.016	0.5	WT	No data
**77**	Dokuz Eylül University Hospital	C1/ G1	No	>64	>16	4	>16	1	0.016	0.016	0.5	WT	Died
**78**	Dokuz Eylül University Hospital	C1/ G1	No	>64	>16	4	>16	1	0.016	0.016	0.5	WT	
**79**	Dokuz Eylül University Hospital	C1/ G1	No	>64	>16	4	>16	1	0.016	0.016	0.5	WT	
**80**	Dokuz Eylül University Hospital	C8/ G86	No	>64	>16	4	>16	1	0.016	0.016	0.5	WT	
**81**	Dokuz Eylül University Hospital	C1/ G4	No	>64	>16	4	>16	1	0.016	0.016	0.5	WT	
**82**	Dokuz Eylül University Hospital	C9/ G93	No	>64	>16	>16	>16	4	0.016	0.016	0.5	WT	Survived
**83**	Dokuz Eylül University Hospital	C9/ G93	No	>64	>16	>16	>16	4	0.016	0.016	0.5	WT	
**84**	Dokuz Eylül University Hospital	C3/ G24	No	>64	>16	1	2	0.125	0.016	0.016	1	WT	Died
**85**	Dokuz Eylül University Hospital	C4/ G39	No	0.25	0.032	0.064	0.064	0.032	0.016	0.016	0.5	WT	Died
**87**	Dokuz Eylül University Hospital	C6/ G57	No	0.25	0.032	0.125	0.032	0.032	0.032	0.016	0.5	WT	Survived
**88**	Dokuz Eylül University Hospital	C6/ G60	No	0.25	0.032	0.125	0.032	0.032	0.032	0.016	0.5	WT	
**89**	Dokuz Eylül University Hospital	C2/ G11	No	0.25	0.032	0.032	0.064	0.032	0.032	0.016	0.5	WT	Died
**90**	Dokuz Eylül University Hospital	C5/ G53	No	0.25	0.032	0.125	0.064	0.032	0.032	0.016	0.5	WT	
**91**	Dokuz Eylül University Hospital	C5/ G46	No	0.25	0.064	0.064	0.125	0.032	0.064	0.016	0.5	WT	Died
**93**	Dokuz Eylül University Hospital	C3/ G27	No	1	0.064	0.125	0.125	0.032	0.032	0.016	0.016	WT	Died
**94**	Dokuz Eylül University Hospital	C2/ G17	≥50%	0.5	0.032	2	4	8	0.016	0.016	0.016	WT	Died
**95**	Dokuz Eylül University Hospital	C3/ G31	≥50%	1	0.032	2	>16	16	0.016	0.016	0.016	WT	
**96**	Dokuz Eylül University Hospital	C3/ G26	≥50%	0.25	0.032	0.125	0.064	0.032	0.032	0.016	0.016	WT	
**97**	Dokuz Eylül University Hospital	C3/ G30	≥50%	0.25	0.032	0.032	0.064	0.032	0.016	0.016	0.016	WT	
**98**	Dokuz Eylül University Hospital	C6/ G59	No	0.25	0.032	0.125	0.064	0.032	0.064	0.016	0.016	WT	

**99**	Dokuz Eylül University Hospital	C6/ G58	No	0.25	0.032	0.125	0.125	0.032	0.125	0.016	0.016	WT	
**100**	Gülhane Training Research Hospital	C4/ G45	No	1	0.25	0.5	1	1	0.064	0.016	0.5	WT	Survived
**101**	Gülhane Training Research Hospital	C1/ G7	No	0.5	0.032	0.032	0.032	0.032	0.125	0.016	0.5	WT	Died
**102**	Gülhane Training Research Hospital	C5/ G52	No	0.5	0.032	0.125	0.032	0.032	0.125	0.016	0.5	WT	
**103**	Gülhane Training Research Hospital	C6/ G56	No	0.5	0.032	0.125	0.032	0.032	0.064	0.016	0.5	WT	Died
**104**	Gülhane Training Research Hospital	C6/ G66	No	0.25	0.032	0.032	0.032	0.032	0.016	0.016	0.5	WT	
**105**	Gülhane Training Research Hospital	C7/ G70	No	0.5	0.064	0.125	0.064	0.032	0.064	0.016	0.5	WT	Died
**106**	Gülhane Training Research Hospital	C2/ G14	No	0.5	0.032	0.125	0.032	0.032	0.032	0.016	0.5	WT	Died
**107**	Gülhane Training Research Hospital	C2/ G18	No	0.25	0.032	0.064	0.064	0.032	0.032	0.016	1	Y221F+ R245K+V362I	Survived
**108**	Gülhane Training Research Hospital	C11/ G123	No	0.25	0.032	0.064	0.032	0.032	0.064	0.016	1	K344N+ V362M	No data
**109**	Gülhane Training Research Hospital	C11/ G124	No	0.5	0.032	0.25	0.032	0.032	0.064	0.016	1	WT	
**110**	Gülhane Training Research Hospital	C3/ G23	No	0.125	0.032	0.125	0.032	0.032	0.032	0.032	1	Y221F+ R245K+K344T+V362I	Died
**111**	University of Health Sciences, Adana City Hospital	C2/ G10	No	0.25	0.032	0.032	0.032	0.032	0.064	0.016	0.5		Survived
**112**	University of Health Sciences, Adana City Hospital	C1/ G8	≥50%	0.25	0.032	0.032	0.032	0.032	0.016	0.032	1	Y221F+ R245K+V362I	Died
**113**	University of Health Sciences, Adana City Hospital	C5/ G54	≥50%	0.25	0.032	0.032	0.064	0.032	0.064	0.032	1	WT	Survived
**114**	University of Health Sciences, Adana City Hospital	C2/ G15	≥50%	0.25	0.032	0.125	0.064	0.032	0.125	0.016	1	WT	Died
**115**	University of Health Sciences, Adana City Hospital	C8/ G82	≥50%	0.25	0.032	0.064	0.064	0.032	0.032	0.016	1	R245K+K344N+V362M	Survived
**116**	University of Health Sciences, Adana City Hospital	C3/ G33	≥50%	0.125	0.032	0.032	0.032	0.032	0.032	0.016	0.5	WT	Survived
**117**	Ondokuz Mayıs University Hospital	C6/ G61	No	0.5	0.032	0.064	0.064	0.064	0.032	0.032	0.5	WT	Died
**118**	Ondokuz Mayıs University Hospital	C4/ G42	No	0.25	0.032	0.032	0.032	0.032	0.064	0.016	0.5	WT	Died
**119**	Ondokuz Mayıs University Hospital	C10/ G108	No	0.25	0.032	0.125	0.064	0.064	0.032	0.016	0.5	WT	Died
**120**	Ondokuz Mayıs University Hospital	C11/ G117	No	0.25	0.032	0.064	0.032	0.032	0.032	0.016	0.5	WT	Died
**121**	Ondokuz Mayıs University Hospital	C11/ G113	No	0.5	0.064	0.064	0.064	0.032	0.064	0.016	0.5	WT	Died
**122**	Ondokuz Mayıs University Hospital	C10/ G102	No	0.5	0.032	0.125	0.064	0.032	0.032	0.016	0.5	WT	Died
**123**	Ondokuz Mayıs University Hospital	C11/ G113	No	0.25	0.032	0.125	0.125	0.032	0.125	0.032	0.5	R245K+V362I	Survived
**124**	Ondokuz Mayıs University Hospital	C11/ G119	No	0.5	0.032	0.064	0.064	0.032	0.064	0.016	0.5	WT	Died
**125**	Ondokuz Mayıs University Hospital	C11/ G112	No	0.25	0.032	0.032	0.032	0.032	0.064	0.016	0.5	WT	Died

**126**	Ondokuz Mayıs University Hospital	C11/ G114	No	0.25	0.032	0.125	0.032	0.032	0.064	0.016	0.5	WT	Died
**127**	Ondokuz Mayıs University Hospital	C11/ G119	No	0.5	0.032	0.125	0.064	0.032	0.032	0.016	0.5	WT	Survived
**128**	Ondokuz Mayıs University Hospital	C10/ G97	≥50%	0.125	0.125	0.064	0.032	0.032	0.016	0.016	0.5	WT	Died
**129**	Ondokuz Mayıs University Hospital	C5/ G51	No	0.5	0.032	0.032	0.032	0.032	0.016	0.016	0.5	WT	Survived
**130**	Ondokuz Mayıs University Hospital	C2/ G19	No	0.125	0.032	0.032	0.032	0.032	0.016	0.016	0.25	WT	Died
**131**	Ondokuz Mayıs University Hospital	C5/ G50	No	0.25	0.032	0.032	0.032	0.032	0.016	0.016	0.25	WT	Died
**133**	Ondokuz Mayıs University Hospital	C2/ G12	≥50%	0.125	0.032	0.032	0.032	0.032	0.016	0.016	0.5	V362I	Died
**134**	Ondokuz Mayıs University Hospital	C2/ G16	≥50%	0.125	0.032	0.032	0.032	0.032	0.016	0.016	0.5	K344N+V362I	
**135**	Ondokuz Mayıs University Hospital	C3/ G22	≥50%	0.5	0.064	0.032	0.032	0.032	0.016	0.016	0.25	WT	Died
**136**	Ondokuz Mayıs University Hospital	C4/ G44	≥50%	0.5	0.032	0.032	0.032	0.032	0.016	0.016	0.25	WT	Died
**137**	Ondokuz Mayıs University Hospital	C3/ G20	≥50%	0.5	0.032	0.032	0.032	0.032	0.016	0.016	0.25	WT	Survived
**138**	Ondokuz Mayıs University Hospital	C8/ G81	No	0.25	0.032	0.032	0.032	0.032	0.016	0.016	0.25	WT	Died
**139**	Ondokuz Mayıs University Hospital	C8/ G90	≥50%	0.25	0.032	0.032	0.032	0.032	0.016	0.016	0.25	WT	Survived
**140**	Erciyes University Hospital	C8/ G84	≥50%	0.25	0.032	0.032	0.032	0.032	0.016	0.016	0.25	WT	Survived
**141**	Erciyes University Hospital	C8/ G76	≥50%	0.25	0.032	0.032	0.032	0.032	0.016	0.016	0.125	WT	Died
**142**	Erciyes University Hospital	C6/ G62	≥50%	0.5	0.032	0.064	0.032	0.032	0.016	0.016	0.5	WT	Died
**143**	Erciyes University Hospital	C8/ G88	≥50%	0.25	0.032	0.032	0.032	0.032	0.016	0.016	0.5	Y221F+ R245K+V362I	Died
**144**	Erciyes University Hospital	C6/ G65	≥50%	0.25	0.032	0.064	0.032	0.032	0.016	0.016	0.5	WT	Survived
**145**	Erciyes University Hospital	C5/ G50	≥50%	0.25	0.032	0.125	0.064	0.032	0.032	0.016	1	WT	Died
**146**	Erciyes University Hospital	C7/ G75	≥50%	0.5	0.064	0.125	0.064	0.032	0.125	0.032	0.5	Y221F+ R245K+V362I	Died
**147**	Erciyes University Hospital	C8/ G76	≥50%	0.25	0.032	0.032	0.032	0.032	0.016	0.016	0.125	WT	Survived
**148**	Erciyes University Hospital	C4/ G38	≥50%	0.25	0.032	0.032	0.032	0.032	0.016	0.016	0.25	WT	Survived
**149**	Erciyes University Hospital	C2/ G13	≥50%	0.125	0.032	0.032	0.032	0.032	0.016	0.016	0.125	WT	Survived
**150**	Erciyes University Hospital	C1/ G6	≥50%	0.125	0.032	0.032	0.032	0.032	0.016	0.016	0.125	WT	Survived
**151**	Erciyes University Hospital	C11/ G122	≥50%	0.5	0.032	0.064	0.032	0.032	0.016	0.016	0.25	WT	Survived
**156**	Trakya University Hospital	C10/ G95	≥50%	0.25	0.032	>16	>16	>16	0.064	0.016	0.5	WT	Survived
**157**	Trakya University Hospital	C3/ G28	≥50%	0.25	0.032	>16	>16	>16	0.016	0.016	0.25	WT	
**158**	Trakya University Hospital	C10/ G99	≥50%	0.25	0.032	>16	>16	>16	0.016	0.016	0.25	WT	Died
**159**	Trakya University Hospital	C6/ G56	≥50%	0.5	0.032	>16	>16	16	0.016	0.016	0.5	WT	Died
**160**	Trakya University Hospital	C11/ G115	≥50%	0.25	0.032	0.032	0.032	0.032	0.064	0.016	0.5	WT	Survived

**161**	Trakya University Hospital	C10/ 107	≥50%	0.5	0.032	0.064	0.064	0.032	0.016	0.016	0.125	WT	Survived
**162**	Trakya University Hospital	C10/ G95	≥50%	0.25	1	0.125	0.064	0.125	0.016	0.016	0.25	WT	Survived
**163**	Trakya University Hospital	C3/ G25	≥50%	0.25	0.25	0.032	0.125	0.064	0.016	0.016	0.25	WT	Survived
**164**	Trakya University Hospital	C8/ G79	≥50%	0.125	0.032	0.032	0.032	0.032	0.064	0.016	0.5	Y221F+ R245K+K344N+V362M	Survived
**165**	Trakya University Hospital	C10/ G105	≥50%	0.25	0.125	0.125	1	0.032	0.016	0.016	0.25	WT	Survived
**166**	Trakya University Hospital	C4/ G35	≥50%	0.5	0.125	0.032	0.125	0.032	0.032	0.016	0.25	WT	Survived
**167**	Trakya University Hospital	C7/ G74	≥50%	0.5	0.032	0.125	0.032	0.032	0.016	0.016	0.25	WT	Died
**168**	Trakya University Hospital	C7/ G71	≥50%	0.25	0.032	0.064	0.064	0.032	0.016	0.016	0.25	WT	Died
**169**	Trakya University Hospital	C1/ G8	≥50%	0.25	0.032	0.032	0.032	0.032	0.016	0.016	0.25	WT	Survived
**171**	Trakya University Hospital	C4/ G36	≥50%	0.25	0.032	0.032	0.032	0.032	0.016	0.016	0.25	WT	Survived
**172**	Trakya University Hospital	C5/ G55	No	>64	0.125	0.064	2	0.125	0.016	0.016	0.25	WT	Survived
**173**	Trakya University Hospital	C9/ G92	No	0.5	0.064	0.064	0.032	0.032	0.016	0.016	0.25	WT	Survived

TABLE S2 | Distribution of genotype clusters among seven hospitals participated in this study.

Centers involved (Isolate number)	Cluster 1	Cluster 2	Cluster 3	Cluster 4	Cluster 5	Cluster 6	Cluster 7	Cluster 8	Cluster 9	Cluster 10	Cluster 11
**DEUH (*n* = 61)**	6	2	9	4	10	8	6	4	4	6	2
**EUH (*n* = 32)**	2	1	0	4	2	2	1	4	1	7	8
**OMUH (*n* = 22)**	0	3	2	2	2	1	0	2	0	5	1
**TUH (*n* = 17)**	1	0	2	2	1	1	2	1	1	5	1
**EUH (*n* = 12)**	1	1	0	1	1	2	1	0	0	0	2
**GTRH (*n* = 11)**	1	2	1	1	1	2	1	0	0	0	2
**UHSACH (*n* = 6)**	1	2	1	0	1	0	0	1	0	0	0

Statistical analysis Section

1) Fluconazole MIC and Cluster association

cluster * FLC_MIC Crosstabulation

Count

		FLC_MIC										
		.032	.125	.250	.500	1.000	2.000	4.000	8.000	32.000	64.000	Total
cluster	C1	0	2	3	2	0	1	0	0	0	4	12
	C2	0	4	4	3	0	0	0	0	0	0	11
	C3	1	2	5	3	2	1	0	0	0	1	15
	C4	1	1	8	2	1	0	0	0	0	0	13
	C5	1	0	6	9	1	0	0	0	0	1	18
	C6	0	0	7	7	2	0	0	0	0	0	16
	C7	0	0	3	7	1	0	0	0	0	0	11
	C8	0	2	10	2	1	0	0	0	0	1	16
	C9	0	0	1	1	0	0	0	1	0	3	6
	C10	0	1	7	8	1	1	1	0	0	2	21
	C11	0	0	12	5	0	0	0	0	1	1	19
Total		3	12	66	49	9	3	1	1	1	13	158

Chi-Square Tests

	Value	df	Asymptotic Significance (2-sided)
Pearson Chi-Square	126.637[a]	90	.007
Likelihood Ratio	96.078	90	.311
Linear-by-Linear Association	.009	1	.925
N of Valid Cases	158		

a. 99 cells (90.0%) have expected count less than 5. The minimum expected count is .04.

Directional Measures

			Value
Nominal by Interval	Eta	cluster Dependent	.313
		FLC_MIC Dependent	.435

.

Ranks

	cluster	N	Mean Rank
FLC_MIC	C1	12	93.79
	C2	11	50.00
	C3	15	76.30
	C4	13	57.42
	C5	18	85.22
	C6	16	84.47
	C7	11	92.95
	C8	16	62.69
	C9	6	125.75
	C10	21	91.43
	C11	19	74.16
	Total	158	

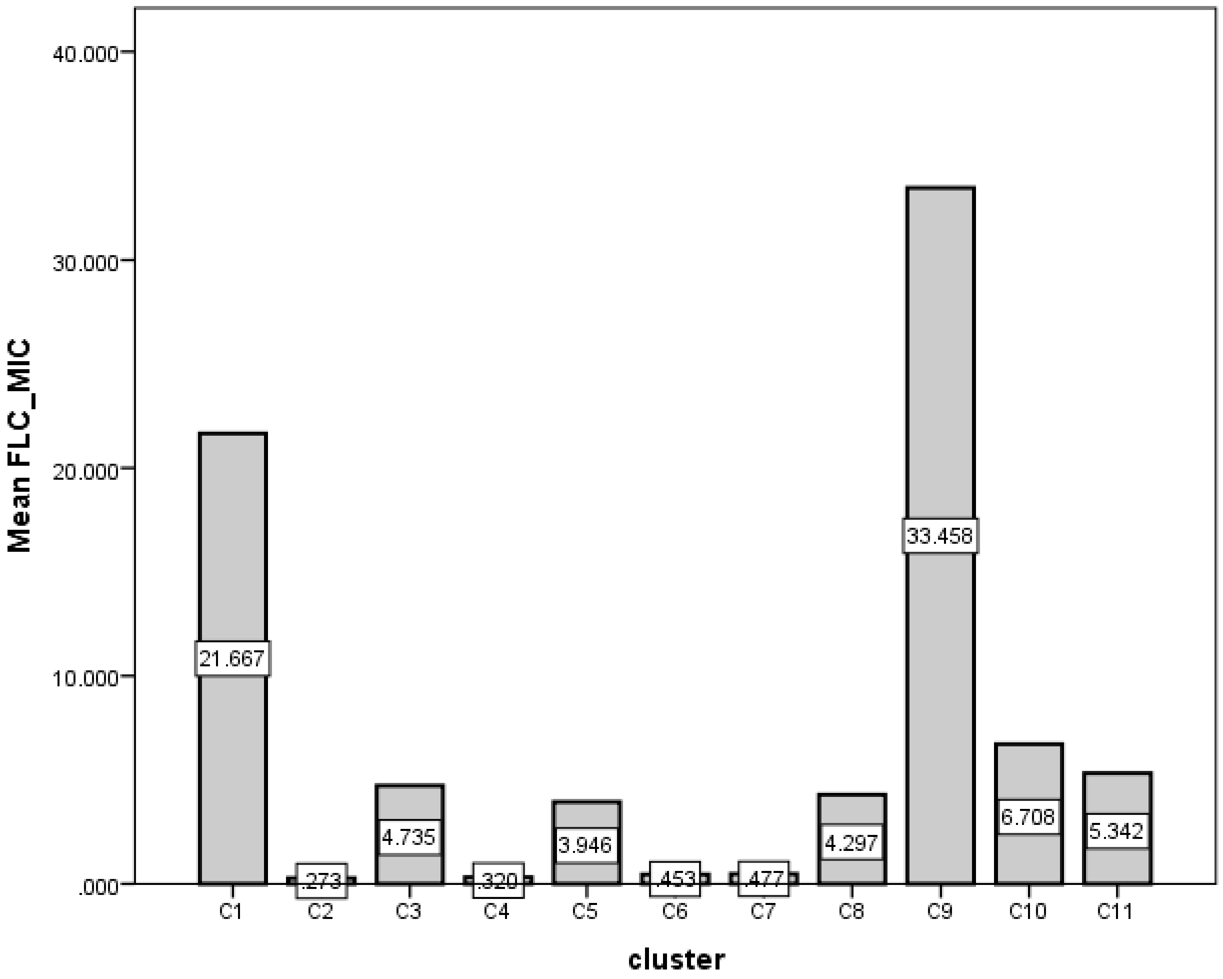

Test Statistics[a,b]

	FLC_MIC
Chi-Square	22.588
df	10
Asymp. Sig.	.012

a. Kruskal Wallis Test

b. Grouping Variable: cluster

C1 * FLC_MIC

Crosstab

Count

		FLC_MIC										Total
		.032	.125	.250	.500	1.000	2.000	4.000	8.000	32.000	64.000	
c_1	.00	3	10	63	47	9	2	1	1	1	9	146
	1.00	0	2	3	2	0	1	0	0	0	4	12
Total		3	12	66	49	9	3	1	1	1	13	158

Chi-Square Tests

	Value	df	Asymptotic Significance (2-sided)
Pearson Chi-Square	17.156[a]	9	.046
Likelihood Ratio	13.129	9	.157
Linear-by-Linear Association	10.359	1	.001
N of Valid Cases	158		

a. 14 cells (70.0%) have expected count less than 5. The minimum expected count is .08.

Symmetric Measures

		Value	Approximate Significance
Nominal by Nominal	Phi	.330	.046
	Cramer's V	.330	.046
N of Valid Cases		158	

C9 * FLC_MIC

Crosstab

Count

		FLC_MIC										Total
		.032	.125	.250	.500	1.000	2.000	4.000	8.000	32.000	64.000	
c_9	.00	3	12	65	48	9	3	1	0	1	10	152
	9.00	0	0	1	1	0	0	0	1	0	3	6
Total		3	12	66	49	9	3	1	1	1	13	158

Chi-Square Tests

	Value	df	Asymptotic Significance (2-sided)
Pearson Chi-Square	41.060[a]	9	.000
Likelihood Ratio	16.847	9	.051
Linear-by-Linear Association	15.206	1	.000
N of Valid Cases	158		

a. 15 cells (75.0%) have expected count less than 5. The minimum expected count is .04.

Symmetric Measures

		Value	Approximate Significance
Nominal by Nominal	Phi	.510	.000
	Cramer's V	.510	.000
N of Valid Cases		158	

2) Cluster and outcome association (0= death and 1= survived)

cluster1 * outcome1 Crosstabulation

Count

		outcome1		Total
		.00	1.00	
cluster1	C1	1	4	5
	C2	5	3	8
	C3	4	4	8
	C4	5	4	9
	C5	8	3	11
	C6	5	3	8
	C7	7	1	8
	C8	7	7	14
	C9	2	2	4

	C10	9	8	17
	C11	9	8	17
Total		62	47	109

Chi-Square Tests

	Value	df	Asymptotic Significance (2-sided)
Pearson Chi-Square	7.887[a]	10	.640
Likelihood Ratio	8.517	10	.578
Linear-by-Linear Association	.006	1	.940
N of Valid Cases	109		

a. 14 cells (63.6%) have expected count less than 5. The minimum expected count is 1.72.

C1 * outcome1

Chi-Square Tests

	Value	df	Asymptotic Significance (2-sided)	Exact Sig. (2-sided)	Exact Sig. (1-sided)
Pearson Chi-Square	2.906[a]	1	.088		

Continuity Correction[b]	1.544	1	.214		
Likelihood Ratio	2.988	1	.084		
Fisher's Exact Test				.163	.108
Linear-by-Linear Association	2.880	1	.090		
N of Valid Cases	109				

a. 2 cells (50.0%) have expected count less than 5. The minimum expected count is 2.16.

b. Computed only for a 2x2 table

C7 * outcome1

Chi-Square Tests

	Value	df	Asymptotic Significance (2-sided)	Exact Sig. (2-sided)	Exact Sig. (1-sided)
Pearson Chi-Square	3.300[a]	1	.069		
Continuity Correction[b]	2.090	1	.148		
Likelihood Ratio	3.794	1	.051		
Fisher's Exact Test				.134	.070
Linear-by-Linear Association	3.270	1	.071		
N of Valid Cases	109				

a. 2 cells (50.0%) have expected count less than 5. The minimum expected count is 3.45.

b. Computed only for a 2x2 table

Model Summary

Step	-2 Log likelihood	Cox & Snell R Square	Nagelkerke R Square
1	140.519[a]	.752	.101

a. Estimation terminated at iteration number 5 because parameter estimates changed by less than .001.

Variables in the Equation

		B	S.E.	Wald	df	Sig.	Exp(B)
Step 1[a]	C1	1.504	1.219	1.522	1	.173	4.500
	C2	-.393	.877	.201	1	.654	.675
	C3	.118	.858	.019	1	.891	1.125
	C4	-.105	.828	.016	1	.899	.900
	C5	-.863	.833	1.073	1	.300	.422
	C6	-.393	.877	.201	1	.654	.675
	C7	1.828	1.174	2.424	1	.020	.161
	C8	.118	.722	.027	1	.870	1.125
	C9	.118	1.112	.011	1	.916	1.125
	C10	.000	.687	.000	1	1.000	1.000
	Constant	-.118	.486	.059	1	.808	.889

a. Variable(s) entered on step 1: C1, C2, C3, C4, C5, C6, C7, C8, C9, C10.

3) Tolerance and outcome association

tolerance * outcome Crosstabulation

Count

		outcome		
		Death	Survival	Total
tolerance	High	16	26	42
	No	52	30	82
Total		68	56	124

Chi-Square Tests

	Value	df	Asymptotic Significance (2-sided)	Exact Sig. (2-sided)	Exact Sig. (1-sided)
Pearson Chi-Square	7.189[a]	1	.007		
Continuity Correction[b]	6.203	1	.013		
Likelihood Ratio	7.216	1	.007		
Fisher's Exact Test				.008	.006
Linear-by-Linear Association	7.131	1	.008		
N of Valid Cases	124				

a. 0 cells (0.0%) have expected count less than 5. The minimum expected count is 18.97.

b. Computed only for a 2x2 table

Symmetric Measures

		Value	Approximate Significance
Nominal by Nominal	Phi	-.241	.007
	Cramer's V	.241	.007
N of Valid Cases		124	

cluster * tolerance3

cluster * tolerance Crosstabulation

Count

		tolerance		Total
		No	High	
cluster	C1	9	3	12
	C2	6	5	11
	C3	6	9	15
	C4	7	7	14
	C5	16	2	18
	C6	13	3	16
	C7	8	3	11
	C8	6	10	16
	C9	6	0	6

	C10	14	7	21
	C11	18	3	21
Total		109	52	161

Chi-Square Tests

	Value	df	Asymptotic Significance (2-sided)
Pearson Chi-Square	26.262[a]	10	.003
Likelihood Ratio	28.296	10	.002
Linear-by-Linear Association	2.431	1	.119
N of Valid Cases	161		

a. 7 cells (31.8%) have expected count less than 5. The minimum expected count is 1.94.

Symmetric Measures

		Value	Approximate Significance
Nominal by Nominal	Phi	.404	.003
	Cramer's V	.404	.003
N of Valid Cases		161	

C3 * tolerance

Chi-Square Tests

	Value	df	Asymptotic Significance (2-sided)	Exact Sig. (2-sided)	Exact Sig. (1-sided)
Pearson Chi-Square	6.029[a]	1	.014		
Continuity Correction[b]	4.685	1	.030		
Likelihood Ratio	5.571	1	.018		
Fisher's Exact Test				.020	.018
Linear-by-Linear Association	5.992	1	.014		
N of Valid Cases	160				

a. 1 cells (25.0%) have expected count less than 5. The minimum expected count is 4.78.

b. Computed only for a 2x2 table

Symmetric Measures

		Value	Approximate Significance
Nominal by Nominal	Phi	.194	.014
	Cramer's V	.194	.014
N of Valid Cases		160	

C5 * tolerance

Chi-Square Tests

	Value	df	Asymptotic Significance (2-sided)	Exact Sig. (2-sided)	Exact Sig. (1-sided)
Pearson Chi-Square	4.027[a]	1	.045		
Continuity Correction[b]	3.021	1	.082		
Likelihood Ratio	4.745	1	.029		
Fisher's Exact Test				.059	.035
Linear-by-Linear Association	4.002	1	.045		
N of Valid Cases	160				

a. 0 cells (0.0%) have expected count less than 5. The minimum expected count is 5.74.

b. Computed only for a 2x2 table

Symmetric Measures

		Value	Approximate Significance
Nominal by Nominal	Phi	-.159	.045
	Cramer's V	.159	.045
N of Valid Cases		160	

C8 * tolerance

Crosstab

Count

		tolerance		
		No	High	Total
C8	.00	103	41	144
	Yes	6	10	16
Total		109	51	160

Chi-Square Tests

	Value	df	Asymptotic Significance (2-sided)	Exact Sig. (2-sided)	Exact Sig. (1-sided)
Pearson Chi-Square	7.678[a]	1	.006		
Continuity Correction[b]	6.191	1	.013		
Likelihood Ratio	7.086	1	.008		
Fisher's Exact Test				.009	.008
Linear-by-Linear Association	7.630	1	.006		
N of Valid Cases	160				

a. 0 cells (0.0%) have expected count less than 5. The minimum expected count is 5.10.

b. Computed only for a 2x2 table

Symmetric Measures

		Value	Approximate Significance
Nominal by Nominal	Phi	.219	.006
	Cramer's V	.219	.006
N of Valid Cases		160	

C9 * tolerance

Crosstab

Count

		tolerance		
		No	High	Total
C9	.00	103	50	153
	Yes	6	0	6
Total		109	50	159

Chi-Square Tests

	Value	df	Asymptotic Significance (2-sided)	Exact Sig. (2-sided)	Exact Sig. (1-sided)
Pearson Chi-Square	2.860[a]	1	.091		
Continuity Correction[b]	1.545	1	.214		
Likelihood Ratio	4.638	1	.031		
Fisher's Exact Test				.178	.099
Linear-by-Linear Association	2.842	1	.092		

N of Valid Cases	159				

a. 2 cells (50.0%) have expected count less than 5. The minimum expected count is 1.89.

b. Computed only for a 2x2 table

Symmetric Measures

		Value	Approximate Significance
Nominal by Nominal	Phi	-.134	.091
	Cramer's V	.134	.091
N of Valid Cases		159	

c11 * tolerance

Crosstab

Count

		tolerance		
		No	High	Total
c11	.00	91	48	139
	Yes	18	3	21
Total		109	51	160

Chi-Square Tests

	Value	df	Asymptotic Significance (2-sided)	Exact Sig. (2-sided)	Exact Sig. (1-sided)
Pearson Chi-Square	3.444[a]	1	.063		
Continuity Correction[b]	2.575	1	.109		
Likelihood Ratio	3.899	1	.048		
Fisher's Exact Test				.079	.049
Linear-by-Linear Association	3.422	1	.064		
N of Valid Cases	160				

a. 0 cells (0.0%) have expected count less than 5. The minimum expected count is 6.69.

b. Computed only for a 2x2 table

Symmetric Measures

		Value	Approximate Significance
Nominal by Nominal	Phi	-.147	.063
	Cramer's V	.147	.063
N of Valid Cases		160	

4) Evaluating the outcome between patients infected with azole-non-susceptible and azole-susceptible isolatess

group * outcome Crosstabulation

Count

		outcome		Total
		death	survival	
group	Azole non-susceptible	11	9	20
	Azole-susceptible	57	47	104
Total		68	56	124

Chi-Square Tests

	Value	df	Asymptotic Significance (2-sided)	Exact Sig. (2-sided)	Exact Sig. (1-sided)
Pearson Chi-Square	.000[a]	1	.987		
Continuity Correction[b]	.000	1	1.000		
Likelihood Ratio	.000	1	.987		
Fisher's Exact Test				1.000	.593
Linear-by-Linear Association	.000	1	.987		
N of Valid Cases	124				

a. 0 cells (0.0%) have expected count less than 5. The minimum expected count is 9.03.

b. Computed only for a 2x2 table

5) Fluconazole/ azole therapeutic failure

Chi-Square Tests

	Value	df	Asymptotic Significance (2-sided)
Pearson Chi-Square	9.233[a]	2	.010
Likelihood Ratio	10.965	2	.004
Linear-by-Linear Association	6.435	1	.011
N of Valid Cases	32		

a. 4 cells (66.7%) have expected count less than 5. The minimum expected count is 1.88.

Block 0: Beginning Block

Variables not in the Equation[a]

			Score	df	Sig.
Step 0	Variables	group1	8.611	1	.046
		group2	5.181	1	.023
		group3	2.876	1	.103

a. Residual Chi-Squares are not computed because of redundancies.

Block 1: Method = Enter

Variables in the Equation

		B	S.E.	Wald	df	Sig.	Exp(B)
Step 1[a]	group1	2.213	1.206	3.365	1	.007	9.143
	group2	22.030	20096.485	.000	1	.029	3692513927.000
	Constant	-.827	.453	3.328	1	.068	.438

a. Variable(s) entered on step 1: group1, group2.

6) Evaluating the expansion of Azole-non-susceptible isolates before and after 2018

Case Processing Summary

	Cases					
	Valid		Missing		Total	
	N	Percent	N	Percent	N	Percent
group * Year	161	100.0%	0	0.0%	161	100.0%

group * Year Crosstabulation

Count

		Year		
		before2018	after 2018	Total
group	Azole non-susceptible	4	16	20
	Azole-susceptible	47	94	141
Total		51	110	161

Chi-Square Tests

	Value	df	Asymptotic Significance (2-sided)	Exact Sig. (2-sided)	Exact Sig. (1-sided)
Pearson Chi-Square	1.439[a]	1	.230		
Continuity Correction[b]	.889	1	.346		
Likelihood Ratio	1.547	1	.214		
Fisher's Exact Test				.308	.174
Linear-by-Linear Association	1.430	1	.232		
N of Valid Cases	161				

a. 0 cells (0.0%) have expected count less than 5. The minimum expected count is 6.34.

b. Computed only for a 2x2 table
